# Successful birth of an IVF baby in a patient with Parkinson's disease

**DOI:** 10.4103/0974-1208.63123

**Published:** 2010

**Authors:** Baxi Asha, Neema Hansali, Pauranik Apoorva

**Affiliations:** Disha Fertility Centre, Saket Nagar, Indore, India; 1 MGM Medical College, Indore, India

**Keywords:** Anti-Parkinsonian drugs, infertility, IVF, levodopa, Parkinson's disease, pregnancy

## Abstract

Parkinson's disease, although rare in young patients, may be encountered in the reproductive age group. We report a rare combination of this disease with infertility, which has not been previously reported. The case record of a 29-year-old woman with infertility and Parkinson's disease are retrospectively reviewed. An IVF indicated for tubal factor infertility resulted in a successful singleton pregnancy. She delivered a healthy male baby without experiencing any worsening of her Parkinsonism. The course of pregnancy remained unaffected by the Parkinson's disease and anti-Parkinsonian drugs. The details of the infertility management, antenatal and postnatal course, and medications are described. With careful evaluation, counseling, and monitoring, IVF may be safely used in women with Parkinson's disease.

## INTRODUCTION

Parkinson's disease, a neurodegenerative disease with a prevalence of 1:1000,[1] generally commences in late life and only 5% of the patients have an onset before the age of 40 years.[2] Hence, the association of Parkinson's disease with the pregnancy is very rare, around only 30 such cases having been reported in the literature.[3] We report an IVF pregnancy in a woman with idiopathic Parkinson's disease presenting with primary infertility.

## CASE REPORT

A 29-year-old lady presented with primary infertility since last 9 years. She had a regular cycle with no menstrual symptoms. She had undergone left salphingoophorectomy 12 years back for a left tubo-ovarian mass causing chronic abdominal pain. The histopathology report was not available. She was diagnosed as suffering from idiopathic Parkinson's disease two years back at the age of 27 years when she had presented to a neurophysician with two additional years of history of gradually progressive bradykinesia, resting tremors, dysarthria, and difficulty in walking. She had good response to levodopa but started experiencing drug-induced dyskinesia within 1 year of therapy. The dose of levodopa was then down-titrated and supplemented by other dopamine agonists. She was on regular medication of bromocriptine 1.25 mg twice daily, levodopa 100 + 25 mg every 4 h, ropinirol 0.5 mg thrice daily, amantadine 100 mg twice daily, and benzhexol 2.0 mg thrice daily. Her Hoehn-Yahr scale of severity was 2 and Unified Parkinson's disease rating score was 90.

The patient was intelligent and cooperative with normal vital signs and unremarkable gynecological examination. Complete infertility workup was done. Hematological screen, hormonal assay, and semen analysis were in the normal range. Prolactin levels were 12 ng/ml (normal range: 5 to 27 ng/ml on day 3 of cycle). Transvaginal sonography showed uterus and right ovary to be normal with ovulation on day 14 of an unstimulated cycle. Hysterosalpingography revealed normal uterus; patent right tube; left tube was not visualized. Laparoscopy revealed dense bowel adhesions on uterus and right adnexa; left tube and ovary was absent. An MRI and CT scan of the brain did not show any abnormality.

Patient and her husband were counseled in detail. With proper consent, an IVF cycle was planned. The long protocol of pituitary downregulation was started in the luteal phase. Four ampoules of Menogen (Ferring, GmbH, Kiel, Germany) were administered after downregulation was achieved. An injection of 10,000 IU of hCG was administered when three follicles measured 18-20 mm. On the day of hCG administration, E2 was 1008 pmol/L, and P 1.98 nmol/L. After 36 h, three oocytes were retrieved transvaginally. Forty-eight hours after retrieval, three embryos were transferred all in four celled stage. Vaginal micronized progesterone 200 mg tid was started from the day of embryo transfer. Twelve days later, serum hCG was 278 IU/ ml. An ultrasound at 6 weeks revealed a live pregnancy. Supportive hormonal therapy was continued till 14 weeks of gestation. The patient successfully continued with the singleton pregnancy till term and did not develop any obstetrical/medical complications throughout the pregnancy. Fetal growth on serial scans was normal. Pregnancy also did not affect the course of Parkinson's disease. Her Hoehn-Yahr and Unified Parkinson's disease rating score did not change before, during, and after pregnancy. The dosage of all the anti-Parkinsonian drugs were increased by 25%, considering the increase in plasma volume and altered pharmacokinetics during pregnancy. Amantadine was the only drug which was stopped because of its teratogenicity.[4] A LSCS was indicated for non reassuring CTG at term and she delivered a healthy male baby with good APGAR scores. Patient had an uneventful postnatal period. After delivery, all the anti-Parkinsonian drugs were continued in the prepregnancy dose. Baby was kept on top feeds due to poor lactation. On serial followup, baby had normal growth and milestones. As the levodopa-induced dyskinesia have always been severe and persistent, patient presently needs assistance of other family members while caring for her baby, lest there be risk of dropping the baby.

## DISCUSSION

Parkinson's disease is a chronic progressive neurodegenerative disease clinically characterized by four cardinal symptoms: Resting tremor, rigidity, bradykinesia, and a characteristic disturbance of gait and posture. The diagnosis is essentially clinical and in this case, the presentation was typical with gradual onset bradykinesia, resting tremors, dysarthria, and difficulty in walking. Also, good therapeutic response to levodopa was the characteristic of Parkinson's disease. Rare metabolic disorders (Wilson's disease, Hallervordan spatz), which may have Parkinsonism features, were not in differential diagnosis and hence extensive laboratory tests were not performed.

Parkinson's disease is rare in young women of reproductive age and no record of successful IVF pregnancy in such patients has been found in the survey of literature. The major issues in such patients are mainly the effect of ovulation induction and pregnancy on the disease, as well as the safety of medication to the fetus during pregnancy and lactation.

The association of Parkinson's disease with infertility seems to be incidental. The pathogenesis of Parkinson's disease is due to loss of dopaminergic neurons in substantia nigra, which does not directly or indirectly contribute to infertility. There are no known contraindications of ovarian stimulation in such patients. The scant existing data suggest minimal effect of Parkinson's disease on pregnancy, childbirth, and neonatal health. There are studies that suggest a possible risk of motor symptoms worsening in connection with pregnancy probably due to multiple influences of sex hormones, estrogens in particular, on basal ganglia function.[4,5] In severe and advanced cases of Parkinson's disease, routine activities may become difficult to perform and might pose greater challenge in pregnancy. No such worsening was noted in our patient and she carried through her pregnancy with remarkable fortitude and family support. All the prescribed anti-Parkinsonian drugs belong to category C (Food and Drug Administration, USA). Previous studies in the literature suggest that there is no or minimal effect of Parkinson's disease on the pregnancy and fetus, and the similar observation was noted in our patient.[5]

The pathogenesis of Parkinson's disease is mainly due to genetic susceptibility to environmental toxins leading to focal dopamine depletion. Genetic changes associated with the environment component are more common in patients with early onset of Parkinson's disease.[6] Dedicated family support is required to ensure quality care for the dependent child and mother. All the above-mentioned issues were counselled in detail with the family and management was accordingly planned.

## CONCLUSION

With careful evaluation, adequate counseling, and regular follow-ups, IVF may be safely used in patients with Parkinson's disease, and collaboration of neurophysician and obstetrician is essential for the management of cases with such a rare neurological disease to ensure the best possible maternal, fetal, and neurological outcome.
